# 

**Published:** 1903-01

**Authors:** 


### LIST OF CONTRIBUTORS TO VOLUME XXIV.

Adams, Dr. G. E.
Anderson, Martha, M.D.
Angle, Edward H., M.D., D.D.S.
Arrington, Dr. B. F.
Baker, George T., D.D.S.
Baldwin, A. E., M.D., D.D.S., LL.B.
Belden, H. E., M.D.
Bogue, E. A., M.D., D.D.S.
Brackett, Charles A., D.M.D.
Brewster, Robert.
Bryan, L. C., D.D.S., F.B.D.C.
Chaddock, Charles G., M.D.
Chittenden, Charles C., D.D.S.
Constant, Thos. E., M.R.C.S.
Curry, John C., D.D.S.
Custer, Dr. L. E.
Davenport, W. S., D.D.S.
Dray, Dr. Arthur R.
Duffield, Dr. Joseph E.
Eaton, Dr. Horace E.
Eglin, Dr. A. C.
Fernald, Adelbert, D.M.D.
Fletcher, M. H., M.D., D.D.S.
Glazier, C. M., D.M.D.
Harrison, Walter, L.D.S. (Eng.), D.M.D. (Harv.).
Heise, O. N., M.D., D.D.S.
Holden, George M., D.M.D.
Hopkins, Samuel A., M.D., D.D.S.
Howe, J. Morgan.
Howe, Percy R., M.A., D.D.S.
Jenkins, N. S., D.D.S.
Luckie, S. Blair, D.D.S.
Kells, C. Edmund, Jr., D.D.S.
Marvel, William W., D.M.D.
McCullough, P. B., D.D.S.
Meriam, Horatio C., D.M.D.
Midgley, Albert L., D.M.D.
Miller, W. D., M.D., D.D.S.
Mills, G. Alden.
Minshall, A. G., M.D.
Neall, Walter H., D.D.S.
Palmer, Dr. J. G.
Parkhurst, Charles E., A.B., D.M.D.
Patten, Charles C., D.D.S.
Piper, James R., D.D.S.
Reeves, W. T., D.D.S.
Rhein, M. L., M.D., D.D.S.
Rollins, William.
Römer, Privatdocent Dr. von.
Ross, Dr. James.
Smith, F. Milton, D.D.S.
Smith, Murdoch C., D.D.S., M.D., D.M.D.
Smith, Dr. T. Victor.
Stanley, Ned A., D.M.D.
Steeves, Alice M., D.D.S.
Stubblefield, D. R., M.D., D.D.S.
Talbot, Eugene S., M.D., D.D.S.
Taylor, Dr. Frank T.
Terrell, George B., D.D.S.
Trostil, H. Elmer, D.D.S.
Weeks, Dr. S. M.
Wentworth, Evan P., D.M.D.
Wetzel, Eugene J., D.M.D.
Wheeler, H. L., D.D.S.

